# The potential of short-wave infrared hyperspectral imaging and deep learning for dietary assessment: a prototype on predicting closed sandwiches fillings

**DOI:** 10.3389/fnut.2024.1520674

**Published:** 2025-01-15

**Authors:** Esther Kok, Aneesh Chauhan, Michele Tufano, Edith Feskens, Guido Camps

**Affiliations:** ^1^Division of Human Nutrition and Health, Wageningen University and Research, Wageningen, Netherlands; ^2^Wageningen Food and Biobased Research, Wageningen University and Research, Wageningen, Netherlands

**Keywords:** hyperspectral imaging, image classification, machine learning, dietary assessment, chemometrics

## Abstract

**Introduction:**

Accurate measurement of dietary intake without interfering in natural eating habits is a long-standing problem in nutritional epidemiology. We explored the applicability of hyperspectral imaging and machine learning for dietary assessment of home-prepared meals, by building a proof-of-concept, which automatically detects food ingredients inside closed sandwiches.

**Methods:**

Individual spectra were selected from 24 hyperspectral images of assembled closed sandwiches, measured in a spectral range of 1116.14 nm to 1670.62 nm over 108 bands, pre-processed with Standard Normal Variate filtering, derivatives, and subsampling, and fed into multiple algorithms, among which PLS-DA, multiple classifiers, and a simple neural network.

**Results:**

The resulting best performing models had an accuracy score of ~80% for predicting type of bread, ~60% for butter, and ~ 28% for filling type. We see that the main struggle in predicting the fillings lies with the spreadable fillings, meaning the model may be focusing on structural aspects and not nutritional composition.

**Discussion:**

Further analysis on non-homogeneous mixed food items, using computer vision techniques, will contribute toward a generalizable system. While there are still significant technical challenges to overcome before such a system can be routinely implemented in studies of free-living subjects, we believe it holds promise as a future tool for nutrition research and population intake monitoring.

## 1 Introduction

Currently most dietary assessment is performed using self-report methods. These consist of recall methods, such as 24-h recalls, the dietary history method, and food frequency questionnaires (1, 2). Yet, also comprise real time monitoring methods, such as food records and duplicate meals. The advantage of self-report methods is that they can give a very detailed overview of a person’s dietary choices. However, the disadvantage of these methods is that they are time-consuming, prone to measurement error and expensive. Furthermore, to a certain extent, recall methods suffer from recall bias, underestimation of intake quantity (1, 2).

Innovations on self-report methods have been made toward web-based and smartphone-based versions of these dietary assessment tools (3). In addition, further automation steps have been developed to reduce measurement errors introduced by manual data entry (4, 5). For instance, machine learning may aid the objective observation of food on a plate by automatic detection of food intake, based on portion size estimation and food identification from images (6–10).

Machine learning applied to color imaging has been highly successful in the automatic detection of the shape, size, color and texture of the world around us (11). Although often limited by the quality of the input data, and the lack of a generalized input dataset, as described by Tahir et al., color images can be used to train algorithms, which can identify food items with increasing reliability (8, 12). The main limitation of using red, blue, green (RGB) color images for the automatic identification of foods comes from some foods being difficult to distinguish visually, because they are similar in color and shape, e.g., black coffee versus cola soda. It is also difficult to see differences in nutritional content of food items within the same category, e.g., full fat cheese versus low fat cheese, just based on the food’s external characteristics such as shape, size, color and texture (8, 12).

Aside from single food items being hard to distinguish, there is an added complexity when the foods are assembled into a home-prepared meal. The most accurate method to determine the nutritional content of a meal is to send it to a lab for testing, which is a common practice in duplicate meal studies (13). Unfortunately, analysis of these duplicates is expensive and does not yield immediate insights due to the processing time of the samples in the lab. Traditional color images often consist of three channels, which represent the reflection of red, green and blue light, in a similar way the human eye sees light. It captures information about an object’s color, shape, size, and texture. The main limitation of using RGB images for intake estimation, is that the nutritional content of the food item cannot be estimated by identifying the type of food alone. RGB image data can be enriched with, for example, the number of calories (14), but caloric information cannot be captured in the shape, size, color and texture of those RGB images. So, while RGB images are good at identifying food items based on those aspects, these types of studies often need additional information, such as recipe and preparation methods, to reliably predict the number of calories in the food item.

In contrast, near infra-red (NIR) spectroscopy can provide information on the chemical composition of food samples, derived from the spectrum of light wavelength reflection, absorption or transmittance. NIR spectroscopy does not provide any spatial information because it is a single point measurement (15). To overcome the limitations of RGB imagery and NIR spectrometry in detecting a combination of chemical composition and spatial characteristics, hyperspectral imaging (HSI) is emerging as a relevant addition to the automatic detection of nutritional intake. Where RGB images capture information on three channels within the visible light range (380–750 nm), hyperspectral images capture a multitude of channels outside the visible light range, giving information about chemical composition using (bio)molecular light reflection, as well as spatial characteristics (shape, size and texture) (16). Thus, when both spatial characteristics and chemical composition need to be considered, HSI is a very suitable method for acquiring data. Within the field of food sciences, HSI in combination with deep learning has successfully been applied to research food quality and safety (17, 18), in clinical settings (19), for food packaging (20), and for monitoring crop health (21–28).

Hyperspectral wavelengths can penetrate deeper into a food product than visible light, depending on the wavelength used and the product analyzed (29, 30). For instance, Lammertyn et al. show in their work a depth of 2-4 mm for apples (29), whereas Arink et al. use tomatoes and conclude a depth of 20 mm (30).The extent to which hyperspectral wavelengths penetrate a product depends on factors such as the product’s properties, thickness, and, as aforementioned, the specific wavelengths used. This unique feature may allow us to capture information beyond surface level, e.g., the content of “closed” foods such as wraps or sandwiches. This advantage is necessary for the improvement of dietary assessment. Currently, for example, open sandwiches can partially be analyzed using traditional color images, which can identify the filling in a similar fashion to other food classification methods (8, 12). However, this is not possible for a closed sandwich. Obtaining an accurate measurement of a closed food would require an individual to “open” the item, so it can be imaged. Since accurate dietary assessment depends on measuring eating habits in free living conditions, eliminating the need to deconstruct “closed” foods just for measuring purposes would increase the accuracy of natural eating behavior measurements.

We aimed to use HSI to determine the fillings in closed sandwiches. A closed sandwich (“double breaded sandwich”) is a staple of the Dutch lunchtime meal, with a high variety of types of bread and fillings to put in between the slices of bread, with or without a layer of butter.

The dataset used in this study consists of hyperspectral images captured from closed sandwiches. Using closed sandwiches as a proof-of-concept for a home-prepared complex meal, this paper analyzes whether machine learning, applied to hyperspectral images, can be a useful methodology to determine the composition of complex meals, and therefore add to the objectiveness of dietary assessment by automatic detection of nutritional intake.

## 2 Materials and methods

### 2.1 Hyperspectral imaging

A hyperspectral image is captured in a so-called hypercube, a 3D structure that represents spatial aspects in 2D (shape, size and texture), e.g., the object it imaged, and per-pixel spectral information in 1D, measuring the interaction (reflectance, transmittance, or absorbance) of NIR light within a sample, to obtain its spectral information (31).

**Figure 1:**
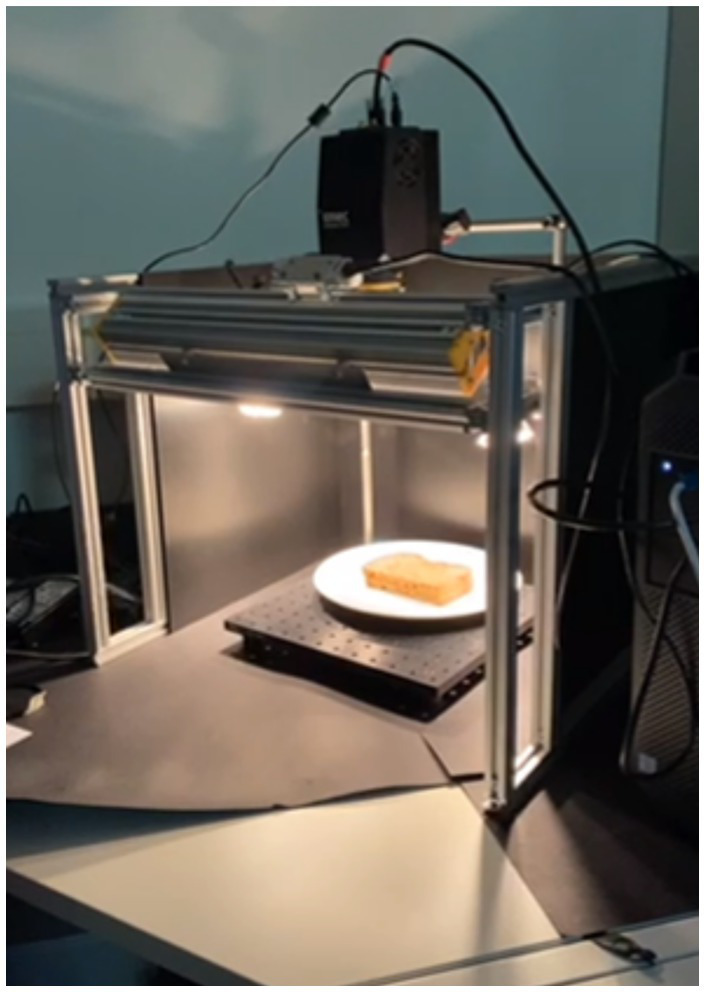
Lab setup; with spectral camera on top, incandescent lighting from four directions, and black cardboard base to minimize light scattering.

This study used an IMEC SWIR Snapscan HSI system (Interuniversity Microelectronics Centre, Leuven, Belgium) with an Optec 16 mm F1.7 SWIR lens (Optec S.p.A., Parabiago, Italy). The full setup can be seen in Figure 1. This is a camera that combines spectral and spatial scanning, and snapshot methods. It captures a spectral range from 1116.14 to 1670.62 nm over 108 bands.

**Figure 2:**
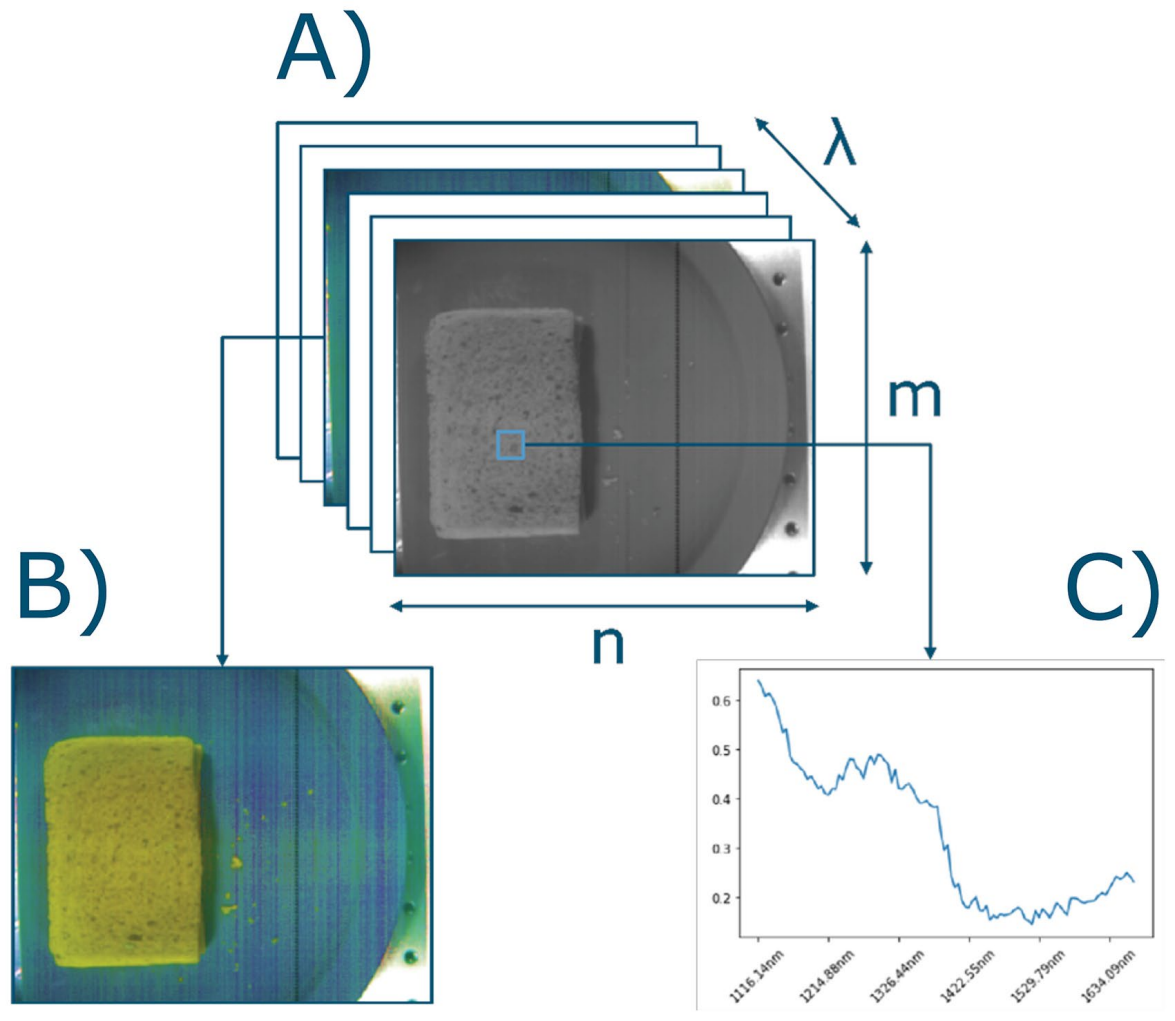
**(A)** Visualized hypercube of a sandwich. Acquired spectral hypercube data is an (n, m, *λ*) hypercube, with n width, m length, λ spectral bands, where each band in the λ dimensions can be visualized as a **(B)** pseudo color image (which band?), and **(C)** mean spectra of selected region.

Spatial scanning can be seen as the scanning part of the hypercube over the total spectral range (*λ*), either by capturing a point (n_i_,m_j_) or a line (n_i_, m_j…k_) of the spatial dimensions (n, m) of the hypercube. Spectral scanning captures the total spatial range (n, m) per slice of the spectral wavelengths, e.g., per band (*λ*_i_). The total dimensions of the hypercube are (n, m, λ). Figure 2 shows a visualization of these dimensions. This notation is used as a reference throughout the article when referring to the dimensions of the data.

### 2.2 Data acquisition

**Figure 3:**
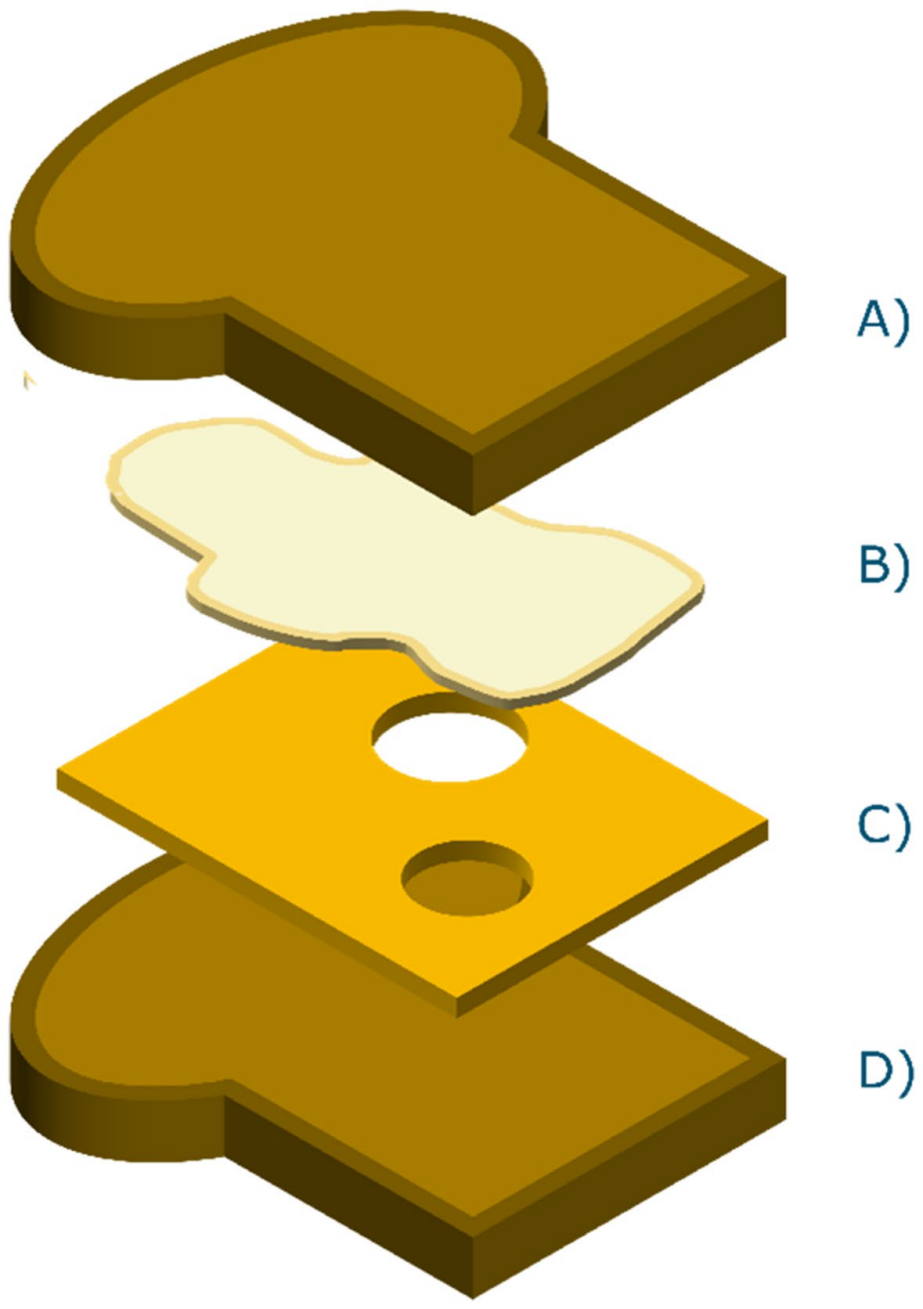
Dutch staple sandwich scheme. **(A)** Top bread slice, **(B)** visualization of evenly spread layer of butter, **(C)** a filling of choice, for example cheese and, **(D)** bottom bread slice.

Due to the lack of publicly available hyperspectral datasets of sandwiches, the choice was made to capture hyperspectral images of sandwiches assembled in-house. The sandwiches were assembled using two types of bread that varied in nutritional content (whole wheat versus white bread). Only one type of butter was used (Albert Heijn brand, 50% full fat butter, with plant-based oils to increase spreadability), which was either added or not (butter/no butter). Six common Dutch types of spreads were selected; jelly (Hero, strawberry flavor), low sugar jelly (Hero, strawberry flavor, reduced sugar), mature cheese (Albert Heijn, Goudse 48+), low fat mature cheese (Albert Heijn, Goudse 30+), peanut butter (Albert Heijn Bio), and chocolate sprinkles (De Ruijter, Milk Chocolate). Figure 3 shows a schematic representation of the layers of an assembled sandwich in the correct order.

All the products were bought from Albert Heijn Zaandam, the largest Dutch supermarket chain, on the day prior to the assembly and data capture. The bread came from the supermarkets’ in-house bakery and was stored in its original plastic packaging. Likewise, the butter, as well as the fillings, were stored in their original packaging, being plastic in the case of the butter and the cheeses, a glass jar in the case of the jelly and the peanut butter, and a cardboard box in the case of the chocolate sprinkles. All products were stored as recommended by the manufacturer. To specify, the butter was stored chilled, all others at room temperature. Additional product details are specified in Supplementary Table S1.

The sandwiches used in this study were assembled based on a study grid with common sandwich characteristics, e.g., a combination of bread, butter and filling. The combination of different breads, butter and filling yielded the grid described in Table 1.

**Table 1:** Sandwich assembly grid to make sandwiches for each of the combinations of conditions.

Sandwich	Filling	Bread type	Butter
A1	Mature cheese	White	No
A2			Yes
A3		Whole wheat	No
A4			Yes
B1	Low fat mature cheese	White	No
B2			Yes
B3		Whole wheat	No
B4			Yes
C1	Jelly	White	No
C2			Yes
C3		Whole wheat	No
C4			Yes
D1	Low sugar jelly	White	No
D2			Yes
D3		Whole wheat	No
D4			Yes
E1	Peanut butter	White	No
E2			Yes
E3		Whole wheat	No
E4			Yes
F1	Chocolate sprinkles	White	No
F2			Yes
F3		Whole wheat	No
F4			Yes

The sandwiches were assembled in order of filling. All components were weighted during assembly. Each sandwich consisted of one slice of bread (35 g) cut in half to become the top and bottom part of the double sandwich. If applicable, butter (5 g) was added to the top slice of bread, in accordance with the grid, and then the filling was added. The amount of filling that was added depended on the type of filling; 15 g jelly, 10 g peanut butter, 10 g chocolate sprinkles or 15 g of cheese. After assembly each sandwich was immediately individually wrapped in plastic foil, to prevent dehydration since moisture is important for accurate NIR measurements (31), and stored at room temperature.

When assembly of all the sandwiches was complete, the sandwiches were moved to the lab where the hyperspectral capturing equipment was set up. The sandwiches were individually unwrapped at the moment of scanning, again to prevent dehydration of the remaining sandwiches, and placed on a plate underneath the camera.

Before scanning the sandwiches, the camera was calibrated with black (98% absorption) and white (95% reflection) reference correction (32). Sandwiches were scanned using the IMEC HSI Snapscan 1.3.8 C100u camera under incandescent lighting conditions, with a black box surrounding the lab setup to limit light scattering outside the study field. The incandescent light brings a near infra-red component to the light, improving the measurements (33). The software used to capture and store the hyperspectral images is the IMEC Snapscan software (version 1.3.0.8, IMEC). The total number of captured hypercubes was 24, as indicated by the number of sandwiches described in Table 1.

### 2.3 Data pre-processing

The data pre-processing pipeline’s main purpose is to reduce noise from the hyperspectral measurements, to select the region of interest (ROI), and to generate features out of this ROI.

Each hypercube consisted of the dimensions (640, 512, 108). The hypercubes were first automatically resized to the dimensions (350, 512, 108), since each of the captured hypercubes contained some artifacts on the discarded part of the image, introduced by the camera. After the first resize, the ROI was automatically selected as a chunk from the middle of the image, resulting in a (100, 200, 108) sized hypercube per capture.

**Figure 4:**
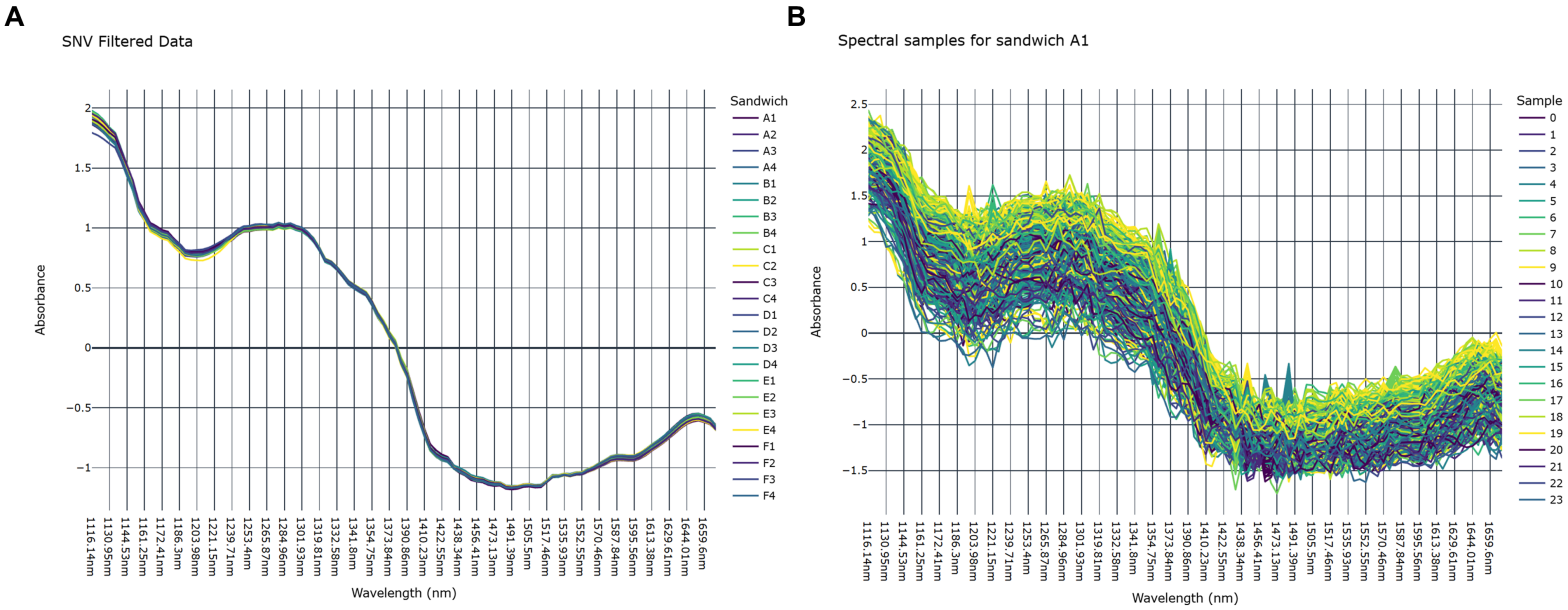
**(A)** Average absorbance pattern per sandwich after SNV transformation, and **(B)** absorbance for an example sandwich (A1), where each sample corresponds with a selected pixel from that hypercube.

To suppress scattering effects in individual hyperspectral images, the spectral responses were filtered using Standard Normal Variate (SNV) transformation (34, 35). In Figure 4A the average wavelength per sandwich after SNV transformation is shown. Figure 4B shows the individual spectral samples for an example sandwich (sandwich A1), to highlight the range of absorbance per sandwich. Each sample in Figure 4B corresponds to a selected pixel from that sandwich using the subsampling strategy.

**Figure 5:**
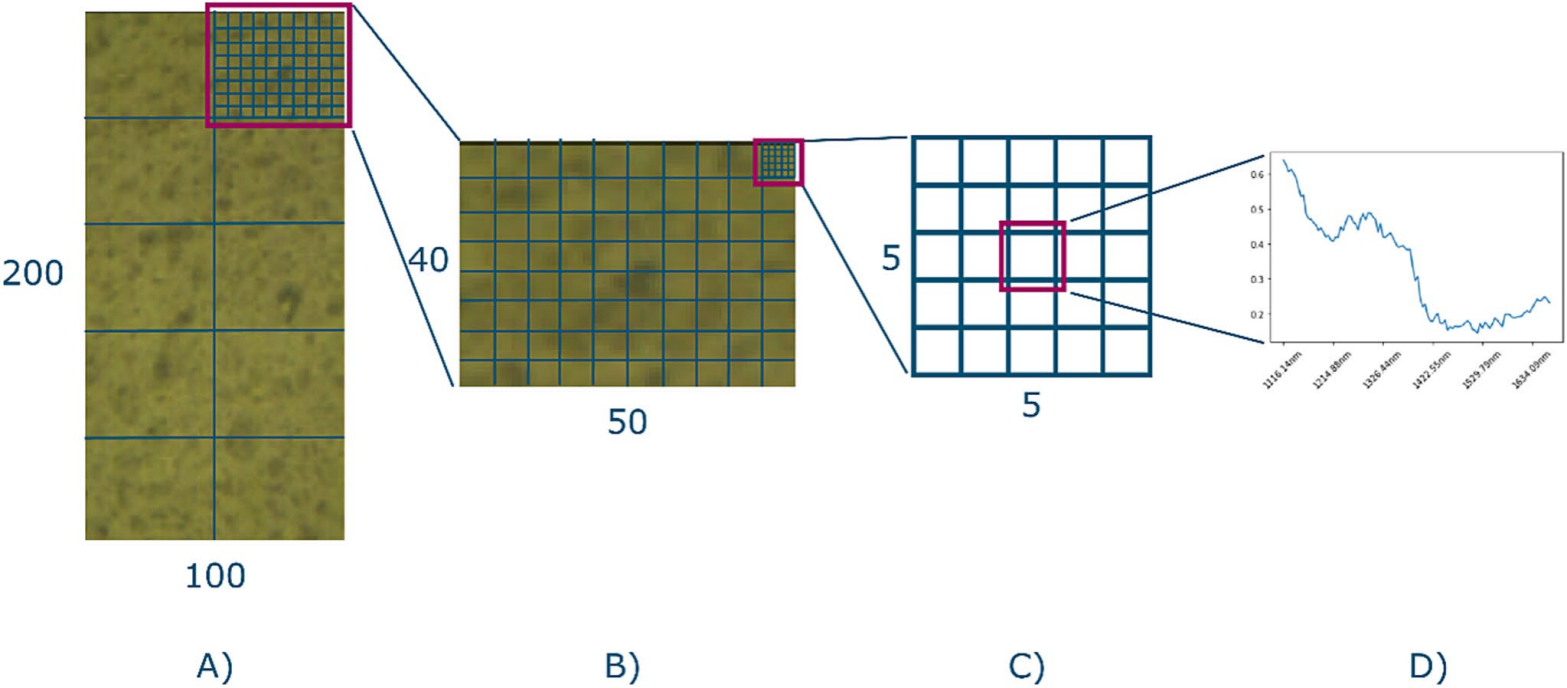
The sampling strategy. **(A)** A given ROI. It is split into 10 equal sized partitions and each partition is randomly assigned to either the train or the test set. **(B)** The single partition is further split into five-by-five sub-grids. **(C)** Center of each sub-grid is selected as the final representation of the spectra of that grid. **(D)** An example spectra.

Subsampling was performed to reduce data interdependency. Adjacent pixels in a hyperspectral image potentially contain information about their neighbor, which will lead to a more optimistic training outcome, if not mitigated. Subsampling was performed by splitting the ROI into partitions, and each partition was assigned to be included as part of the model training, i.e., the train set, or model evaluation, i.e., the test set which was included as a split label. Figure 5 shows a schematic representation of the process. Each ROI (100, 200, 108) was split into 10 partitions (see Figure 5A) of dimensions (50, 40, 108), after which each partition was randomly assigned as part of the train or test set based on a 70–30 split ratio. The subsampling strategy therefore causes 70% of each hyperspectral image to be used for training, and 30% for testing. This also means that the bread, butter, and filling characteristics, i.e., the ground truths about each sandwich, were equally represented in both the train and test set.

Samples were then extracted based on a five-by-five grid subdivision of the partition (see Figures 5B,C), where the center of each grid was included as the final selected sample (see Figure 5D). This yielded 800 samples per image, with a total of 19,200 spectral samples of 108 bands, of which 13,440 were assigned to the train set, and 5,760 to the test set. Thus, effectively unfolding the 24 hypercubes into a data frame with 19,200 rows and 108 columns.

For each sample additional features were generated from first, second and third derivatives. The total dimensions of the dataset were increased to 426 variables and 19,200 samples. Each of these samples was automatically labeled based on the sandwich grid (Table 1), which was done by string parsing the filenames of the scanned hypercubes and matching those to the encoded sandwich names in the grid.

The final ground truth labeling resulted in a multi-class multi-output classification problem, which was reduced to a multi-class single label problem by training three separate models to predict bread type (white/wheat), presence of butter (yes/no), and filling type (mature cheese/low fat cheese/jelly/low sugar jelly/peanut butter/chocolate sprinkles).

### 2.4 Model training

Using the Python programming language (version 3.9) multiple algorithms were trained on the data. Partial Least Squares Discriminant Analysis (PLS-DA) was chosen as a baseline algorithm, since it is well established in the field of HSI and NIR spectroscopy analysis (31) The implementation from the pychemauth library (36) was used. Additional classical machine learning algorithms were trained using the pycaret library (37), and a Multi-Layer Perceptron (MLP) was trained using the Scikit-learn library (38). Since the dataset contains three target characteristics that we would like to predict, i.e., bread, butter and filling, an model was trained for each of these targets. The choice for three models was made deliberately, since a transparent evaluation was needed. If a model were to be trained on all three targets simultaneously, a so-called multi-class multi-output classification, it would be less straightforward to generate insights on where an eventual misprediction occurred. On multi-class multi-output classifications, traditional confusion matrix-based evaluation metrics cannot be applied. Therefore, the decision was made to create a single model for each target, so the evaluation can give insight into what parts of the sandwich were poorly predicted by the algorithm.

PLS-DA was initialized based on the number of components, being 150 components for the “bread” model, 150 components for the “butter” model, and 300 components for the “filling” model, which were optimized based on the accuracy score.

Five-fold cross validation (CV) was used to determine the best performing classical machine learning model using pycaret.

The MLP model was initialized with parameters for four hidden layers, with decreasing sizes of 322, 218, 108, and 54 nodes. Based on current practices in the field we chose an adam solver and a relu activator as additional parameters for the hidden layers of the model (39, 40). The learning rate, e.g., how fast the model adapts to the problem at hand, was set to 0.001. The model was trained over a maximum of 1,000 epochs, meaning the full training data passes through the network at maximum 1,000 times, and minimized the log loss score. Models stopped training if the accuracy on the interval validation set did not improve over 50 epochs with a tolerance of 0.000010.

CV using grid search was used to establish the best topology for the MLP. The parameters that were optimized were hidden layer sizes, solver, activator, and learning rate. The grid from Table 2 shows the different options for each of these parameters. The CV grid search yielded a total of 88 combinations of parameters for each target variable. CV was performed in 5-fold, so each target variable was trained for 440 models in total during the cross-validation phase. The three models were then asked to predict the sandwich characteristics using the test set. For each model the output was evaluated using a confusion matrix for the test set predictions, as well as a per model plot of the accuracy and log loss score per epoch during training.

**Table 2:** Grid search CV parameters for the MLP. Each parameter combination yields a separate model to be cross validated.

Learning rate	Solver	Activation	Hidden layer sizes
0.001	Adam	Relu	(218)
0.01	Sigmoid	Tanh	(427)
			(218, 108)
			(256, 128)
			(512,256)
			(218, 108, 54)
			(512, 256, 128)
			(640, 427, 213)
			(322, 218, 108, 54)
			(512, 256, 128, 64)
			(640, 427, 213, 107)

The confusion matrix gives insight into the trade-off between predicted classes compared to the actual classes. This trade-off is captured as the number of true positives (TP), true negatives (TN), false positives (FP), and false negatives (FN). Multiple performance metrics can be calculated using these numbers. Accuracy is defined in Equation 1 as:


(1) $$\mathrm{Accuracy}=\frac{1}{N}\overset{N}{\underset{i=1}{\sum }}\frac{TP_{i}+TN_{i}}{TP_{i}+TN_{i}+FP_{i}+FN_{i}}$$

Where N is the total number of classes, and $TP_{i}$ is the number of true positives for class i , similarly for true negatives, false positives and false negatives. In other words, the total number of correct predictions is divided by the total number of predictions in the test set (41).

The accuracy over epochs plot during training is not calculated by the test set, but by the internal validation set, which is a subset of the training set, randomly selected by the algorithm itself. While this dataset is used during training, the idea is that the plot gives insight into how well the model is learning over time, and whether the learning has plateaued.

The log loss score is calculated to assess the certainty of the classification decision made by the model. Log loss offers a more nuanced performance metric than accuracy. Using both accuracy and log loss allows for an evaluation of performance through the number of correct predictions and the model’s confidence in these predictions. Log loss can be defined in Equation 2 as:


(2) $$\log\;\mathrm{Loss}=-\frac{1}{N}\overset{N}{\underset{i=1}{\sum }}\overset{C}{\underset{j=1}{\sum }}y_{ij}\log\left({\hat{y}}_{ij}\right)$$

Where N is the number of samples, C is the number of classes, $y_{ij}$ is the indicator whether class j has been correctly classified for sample i , and ${\hat{y}}_{ij}$ is the predicted probability that sample i belongs to class j . In other words, the log loss score is the average negative log-likelihood of the observed classes given the predicted probabilities. Predictions that are confident but incorrect get a higher penalty in this score, and the aim of the MLP is to minimize this score as close to 0 as possible.

## 3 Results

To assess whether machine learning applied on HSI could predict the components of a closed sandwich, multiple models were trained on a set of 24 sandwich HSI’s. A baseline PLS-DA model was trained, several classical machine learning models and an MLP. Three targets were learned in individual models; to predict type of bread, presence of butter, and type of filling.

The training of the PLS-DA resulted in overall accuracy scores of ~73% for the bread model, ~58% for the butter model, and ~ 17% for the fillings model. The results are shown in Supplementary Table S2.

The training of the pycaret models, using CV, yielded different predictors for the bread, butter and filling targets. The bread target resulted in a linear regression model with an accuracy of ~73% on the test set. Similarly, the butter target training resulted in a linear regression model, yet with a ~ 57% accuracy on the test set. The filling target resulted in a ridge classifier, with an accuracy of ~28%. The total confusion matrix results can be found in Supplementary Table S3.

The hyperparameter tuning of the MLP resulted in a topology of four hidden layers (322, 218, 108, 54), an adam solver, relu activator and 0.001 learning rate, with an average accuracy score for the bread model of ~80%, an accuracy of the butter model of ~60%, and for the filling model ~24%. The total confusion matrix with the MLP results can be found in Supplementary Table S4.

**Figure 6:**
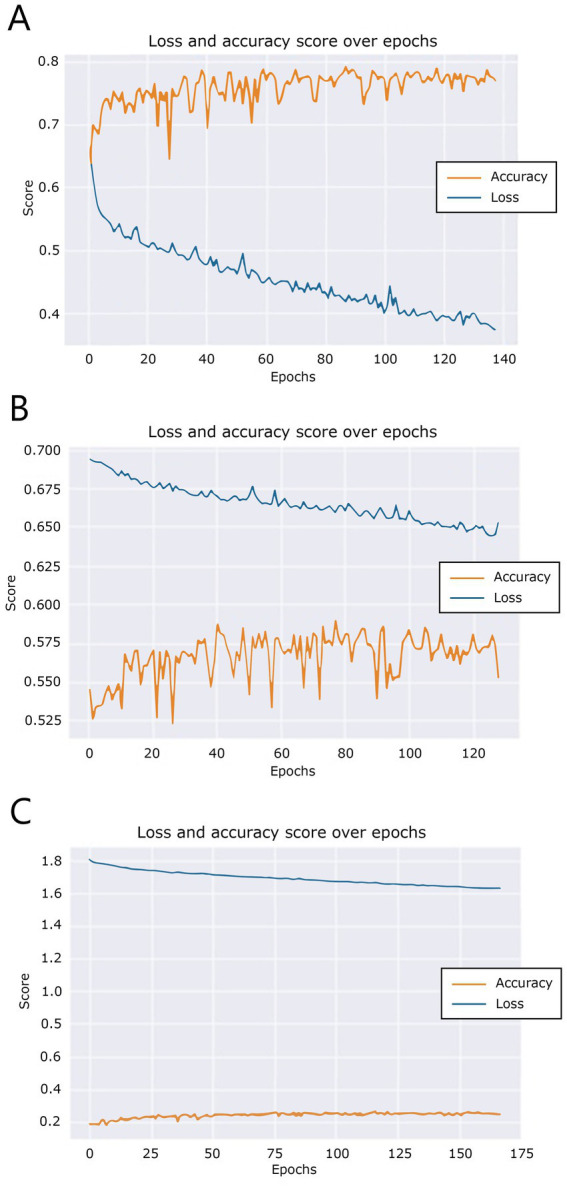
Training and validation loss scores over epochs for **(A)** the “bread” model, **(B)** the “butter” model, and **(C)** the “filling” model.

During training, over the course of the epochs, the model generated training accuracy and loss data. These data are visualized in Figure 6. Figure 6A shows that the accuracy of the bread model does not improve overall after 88 epochs, triggering the training auto stop after epoch 138. A similar pattern can be seen in Figure 6B, where the butter model training did not improve after 79 epochs, so at epoch 129 training was stopped. Finally, Figure 6C shows that training of the fillings model did not improve after epoch 113, triggering the auto stop after epoch 163.

To provide additional insight into the performance of the individual classes, the confusion matrix, showing the frequency of each prediction, can be found in Table 3. This is a combination of each of the best performing models for each target. The results in this table show that the MLP performs best in predicting the classification outcomes for bread and butter targets, where the ridge classifier performs best in predicting the outcome for the filling target. The evaluation on unseen test data show that relevant information in the spectra of the HSI allow for prediction of bread type, butter presence and filling type.

**Table 3:** Confusion matrix containing the prediction results of the best performing models from each target. The bread and butter results from the MLP, the filling results from the ridge classifier.

			Predicted									
			Bread		Butter		Filling					
			White	Whole Wheat	No	Yes	Mature cheese	Low fat mature cheese	Jelly	Low sugar jelly	Peanut butter	Chocolate sprinkles
Ground truth	Bread	White	**0.81**	0.19								
		Whole wheat	0.21	**0.79**								
	Butter	No			**0.60**	0.40						
		Yes			0.41	**0.59**						
	Filling	Mature cheese					**0.28**	0.13	0.17	0.07	0.15	0.21
		Low fat mature cheese					0.27	**0.15**	0.09	0.06	0.21	0.23
		Jelly					0.18	0.10	**0.24**	0.09	0.11	0.28
		Low sugar jelly					0.19	0.14	0.17	**0.11**	0.13	0.26
		Peanut butter					0.15	0.12	0.09	0.07	**0.33**	0.24
		Chocolate sprinkles					0.11	0.08	0.12	0.04	0.10	**0.56**

Highlighted in bold are the correct predictions.

Table 3 shows that most of the mispredictions fall within the fillings category. Mispredictions occurred more frequently between the different sugary fillings (jelly, low sugar jelly, chocolate sprinkles), and between the different fatty fillings (mature cheese, low-fat mature cheese, peanut butter). Some mispredictions seem to also stem from texture morphology of the filling.

## 4 Discussion

The focus of this work was a proof-of-concept test to assess whether machine learning, applied to hyperspectral images, could add to the current dietary assessment toolkit. Using sandwiches as a test food, we hypothesized that the addition of HSI could improve dietary assessment, because both NIR and SWIR wavelengths could be used to look further than the top layer of the sandwich and detect what filling(s) were in between the slices of bread. The algorithms that were trained in this work performed well in the classification of the bread type, with an average accuracy score of ~80%, which was expected given that the bread is the first layer of the sandwich the light interacts with. The model predicted the presence of butter with a ~60% accuracy, which is above random assignment of labels, but leaves substantial room for improvement. The average accuracy score of the prediction of a filling was ~28%, which is somewhat higher than a random chance for this six class multi-class classification. This also implies that the model can find relevant information in the spectra to predict the filling. CV with 5 folds drove the selection of best performing model, selected based on accuracy of the validation set, both for the classical machine learning models and the MLP. Overall, while there is still considerable room for improvement, the combination of technologies seems promising.

While a generalized input dataset should consist of more than sandwiches, the sandwiches used in the current study consisted of a combination of multiple food products and serve as a starting point for this proof-of-concept. The fillings in between the bread were of similar thickness creating layers from the top of the sandwich all the way down to the plate, meaning that the profile of the layers looks similar, except for the chocolate sprinkles. This homogeneity yields similar wavelength profiles for each of the pixels in the ROI, especially when looking at the spectra for a single sandwich (see Figure 2). Similar data could be captured using a single point measuring technique such as NIR. While a NIR sensor has a single point measurement, which generates a single spectrum, an advantage of using HSI is that it captures multiple measurements in a matter of seconds, generating a significantly larger body of measurements to train an algorithm on. However, HSI is most useful when applied to non-homogeneous samples consisting of multiple food items where spatial aspects need to be considered, such as a salad or a plate of breakfast items, or when it is looking at the composition of a food item it its entirety, rather than a single sample (42, 43).

Spatial aspects become important when we want to use this technology for full dietary assessment, because the nutritional content of the plate alone is not sufficient to estimate nutritional intake. For full dietary assessment, the weight or volume of the food is needed (44). A setup that would combine volumetric information with the data of the HSI to determine composition, should be able to help achieve this. There is already evidence showing HSI combined with machine learning can be used to detect the difference in similar looking food items (45). Boosted by segmentation, the individual food items in combined foods can be detected (42). However, these types of systems are based on classification of the food item itself, e.g., each food item is assigned a label in the target set. Since the cardinality of the set of food items to be classified is potentially infinite, the required dataset to train such an algorithm becomes infinitely large as well. Therefore, the spatial component of the HSI will also be interesting for segmentation of the image into similar areas, and estimation of macronutritional content of these separate items that comprise the combined foods, such as tomatoes and croutons in salads. HSI, and similar NIR and SWIR sensors related techniques, are often used in the field of nutrition to help analyze food quality and safety in specific tasks, such as described by Feng and Sun and Park et al. (16, 17). Limited literature is available on the analyzes of food composition from a generalized input dataset, only for specific target problems and food groups (17, 40–42).

We acquired data from 24 sandwiches in a grid-based composition to ensure a balance of the target variable (see Table 1), but prepared only one sandwich sample per composition. Therefore, we captured only one hyperspectral image per sandwich composition. The model was trained and tested on subsamples from the ROI of the image. Since we did not prepare a second set of sandwiches to test the model, there is an interdependency within the spectra samples. We partially mitigated this by selecting subsamples with a range of pixels surrounding the sample, so there is no direct interdependency based on neighboring pixels, but the effect could still be present and partially explain the results of the test model. In future studies additional sandwiches should be assembled per type, so that the spectra samples in the test data would be independent from those in the training data, and therefore be a better representation of the true performance of the model.

In addition, the data acquisition of multiple sandwiches per type, while minimizing data interdependency, implies a larger storage volume. The images captured in this study required around 138 MB of storage per image. This adds up quickly for the storage of raw data. This also includes datapoints we do not need, i.e., the pixels of the image that contain the plate the food is resting on. Future research should study the relevant wavelengths in each image, so that these wavelengths can be the determining factors for the camera used to capture the data, and reduce the storage required.

SNV filtering, as well as derivative analysis, is a common way to pre-process the data from hyperspectral images. It corrects lighting related issues that are caused by the morphology of the food item (46). These filtering steps possibly have transformed the data in such a way that the detection of butter and fillings below the surface were impacted, considering other research in the same field does not apply these filtering steps in the same order (47). The camera used for data acquisition captures a spectral range from 1116.14 nm to 1670.62 nm, which should be sufficient to detect the composition of the separate sandwich elements based on descriptions from previous work (47). The wavelengths measured by the camera may not be enough to reliably penetrate the lower layers of the bread, therefore no data of light being reflected by the filling is captured. A camera with additional detection range (e.g., outside the range of 1116.14–1670.62 nm used in this study) may yield different measured wavelengths and could provide additional information.

The topology of the MLP which performed best in the classification of the bread and butter targets contained four layers of reducing size. The number of layers and their sizes was manually decided upon. While selected by the CV phase, their inclusion in the CV grid was a manual process, with variation to the depth and width added to allow for classification based on more complex features. The selection of a four hidden layer based topology during CV suggests that the shallow networks fail to capture an underlying non-linear relationship in the data. The width of the layers was based on a reducing size, common in convolutional architectures, to reduce computational efforts (48).

There are different approaches to finding the optimal number of hidden layers and nodes, such as a model based approach instead of a grid search. The aim of this study was not to find an optimal topology, but to evaluate if the combination of HSI and ML could work to detect sandwich fillings. However, continuing the work with a different methods to determine the number of hidden layers and the width of the hidden layers would help establish a more structured approach to finding an optimal topology (49).

Looking at the results in Table 3, we see that one of the problem areas in the fillings is the prediction of low sugar jelly versus regular jelly. Similarly, we see a problem area in the prediction of low-fat mature cheese versus mature cheese and peanut butter. Not all the fillings have a higher than random prediction rate, meaning that the model can find relevant information in the spectra to predict some of the fillings, but it is possible that the model has trouble distinguishing them based on nutritional content, or based on the texture morphology of the filling. If the incandescent light does not interact reliably with the lower layers at all, due to limitations from the spectral range or physical aspects of the food, we need to consider that the model is learning based on some aspects in the noise or scattering effects, since we have an interdependency in our data. To test whether the model is predicting based on morphology, a future experiment could measure sandwiches with fillings that have similar nutritional content, but different textures, versus sandwiches with similar textured filling but different macronutrient composition. This experiment should also include double sampling, to make sure there is no interdependency in the data.

The current setup of classifying the types of bread, butter, and fillings has the limitation that, while each element has a distinct composition that is detectable in the spectra, the true composition of food can only be detected using a duplicate meal approach (2). Including multiple samples per distinct plate of food, as well as measuring the exact composition of the meal using duplicate meals, would help create a finite multilabel regression task for future prediction of composition instead of classification of individual food items, which is an open set classification problem. By training an algorithm to estimate the macronutrient composition of the food, the output of the algorithm is reduced to a regression on a small number of target labels. This would also help to narrow down the required wavelengths, because it can be analyzed which set of wavelengths corresponds to which macronutrient.

Lastly, there are multiple potential methods to narrow down the required wavelengths, for example using k-nearest neighbors (50), derivative based analysis (43), or partial least square regression (42). Some of the works mentioned describe the relationship between a certain macronutrient and the corresponding absorption band, e.g., 1,215 nm is described as the absorption band of fat by ElMasry et al. (42). Similarly, Benes et al. describes very extensively the wavelength at which a certain macronutrient can be measured, but in a specific set of food items, namely powdered snack products (47). Reducing the number of wavelengths that need to be acquired will reduce the complexity of the required training data set, and the outcome, as well as create possibilities for additional pre-processing and filtering methodologies, such as PCA assisted segmentation (42, 43). Many food items “in the wild” ought to be tested before a comprehensive conclusion can be made regarding the set of wavelengths applicable for the measurement of a certain macronutrient, and thus which features are needed as input to the model. In the end, the goal is to improve dietary assessment in such a way that we can link the food composition on the plate detected by HSI to our food diaries based on the Dutch Food Composition Database (NEVO) (51).

## 5 Conclusion

This work describes a prototype to demonstrate and assess HSI and machine learning as a candidate future tool for dietary assessment. The initial results of prediction accuracy of ~80% for the bread target, ~60% for the butter target, and ~ 28% for the fillings target, combined with a clear potential for improvements in the methodology with regards to model topology and parameters indicate this warrants further exploration for prediction of meal composition of home-made complex meals.

When reliably built, a machine learning based system that utilizes hyperspectral images to detect composition of combined foods, together with a system that can reliably estimate weight and volume of the food, could be used to improve and support dietary assessment. This way, we can have more accurate measures of dietary intake in a free living population, improving the quality of dietary exposure assessment and monitoring of intakes for research of public health.

## Data Availability

The datasets presented in this study can be found in online repositories. The names of the repository/repositories and accession number(s) can be found at: https://www.researchgate.net/publication/381741409_Hyperspectral_Sandwich_A1_IMEC_SnapScan.

## References

[1] Burrows TL Ho YY Rollo ME Collins CE. Validity of dietary assessment methods when compared to the method of doubly labeled water: a systematic review in adults. Front Endocrinol. (2019) 10:850. doi: 10.3389/fendo.2019.00850, PMID: 31920966 PMC6928130

[2] Shim J-S Oh K Kim HC. Dietary assessment methods in epidemiologic studies. Epidemiol Health. (2014) 36:e2014009. doi: 10.4178/epih/e2014009, PMID: 25078382 PMC4154347

[3] Brouwer-Brolsma EM Lucassen D de Rijk MG Slotegraaf A Perenboom C Borgonjen K. Dietary intake assessment: from traditional paper-pencil questionnaires to technology-based tools In: Athanasiadis IN Frysinger SP Schimak G Knibbe WJ, editors. Environmental software systems. Data science in action. IFIP advances in information and communication technology. Cham: Springer International Publishing (2020). 7–23.

[4] Cade JE. Measuring diet in the 21st century: use of new technologies. Proc Nutr Soc. (2017) 76:276–82. doi: 10.1017/S0029665116002883, PMID: 27976605

[5] Zhao X Xu X Li X He X Yang Y Zhu S. Emerging trends of technology-based dietary assessment: a perspective study. Eur J Clin Nutr. (2021) 75:582–7. doi: 10.1038/s41430-020-00779-0, PMID: 33082535

[6] Chen HC Jia W Li Z Sun YN Sun M. 3D/2D model-to-image registration for quantitative dietary assessment: 38th annual northeast bioengineering conference, NEBEC 2012. 2012 38th Annual Northeast Bioengineering Conference, NEBEC 2012. IEEE (2012) 95–96. 10.1109/NEBC.2012.6206979 PMC4058710 24944506

[7] Chen M Dhingra K Wu W Sukthankar R. PFID: Pittsburgh fast-food image dataset. 2009 16th IEEE International Conference on Image Processing (Cairo, Egypt: ICIP) (2009) 289–292.

[8] He J Shao Z Wright J Kerr D Boushey C Zhu F. Multi-task image-based dietary assessment for food recognition and portion size estimation. In 2020 IEEE Conference on Multimedia Information Processing and Retrieval (MIPR). IEEE. (2020) 6:49–54.

[9] Hafiz R Islam M Khanom R Uddin MS. Image based drinks identification for dietary assessment (2016):192. doi: 10.1109/IWCI.2016.7860364,

[10] Lo FP-W Sun Y Qiu J Lo B. A novel vision-based approach for dietary assessment using deep learning view synthesis. 2019 IEEE 16th International Conference on Wearable and Implantable Body Sensor Networks (BSN). IEEE (2019). p. 1–4.

[11] Voulodimos A Doulamis N Doulamis A Protopapadakis E. Deep learning for computer vision: a brief review. Comput Intell Neurosci. (2018) 2018:e7068349:1–13. doi: 10.1155/2018/7068349, PMID: 29487619 PMC5816885

[12] Tahir GA Loo CK. A comprehensive survey of image-based food recognition and volume estimation methods for dietary assessment. Healthcare. (2021). 9:1676. doi: 10.3390/healthcare9121676 PMC8700885 34946400

[13] Trijsburg L de Vries J Boshuizen HC Hulshof PJM Hollman PCH van 't Veer P. Comparison of duplicate portion and 24 h recall as reference methods for validating a FFQ using urinary markers as the estimate of true intake. Br J Nutr. (2015) 114:1304–12. doi: 10.1017/S0007114515002871, PMID: 26314241

[14] Darapaneni N Singh V Tarkar YS Kataria S Bansal N Kharade A. Food image recognition and calorie prediction. 2021 IEEE International IOT, Electronics and Mechatronics Conference (IEMTRONICS), IEEE. (2021). p. 1–6.

[15] Cen H He Y. Theory and application of near infrared reflectance spectroscopy in determination of food quality. Trends Food Sci Technol. (2007) 18:72–83. doi: 10.1016/j.tifs.2006.09.003

[16] Chen Y-R Chao K Kim MS. Machine vision technology for agricultural applications. Comput Electron Agric. (2002) 36:173–91. doi: 10.1016/S0168-1699(02)00100-X

[17] Feng Y-Z Sun D-W. Application of hyperspectral imaging in food safety inspection and control: a review. Crit Rev Food Sci Nutr. (2012) 52:1039–58. doi: 10.1080/10408398.2011.651542, PMID: 22823350

[18] Park B Shin T-S Cho J-S Lim J-H Park K-J. Characterizing hyperspectral microscope imagery for classification of blueberry firmness with deep learning methods. Agronomy. (2022) 12:85. doi: 10.3390/agronomy12010085, PMID: 39659294

[19] Thiem DGE Römer P Gielisch M Al-Nawas B Schlüter M Plaß B. Hyperspectral imaging and artificial intelligence to detect oral malignancy – part 1 - automated tissue classification of oral muscle, fat and mucosa using a light-weight 6-layer deep neural network. Head Face Med. (2021) 17:38. doi: 10.1186/s13005-021-00292-0, PMID: 34479595 PMC8414848

[20] Medus LD Saban M Francés-Víllora JV Bataller-Mompeán M Rosado-Muñoz A. Hyperspectral image classification using CNN: application to industrial food packaging. Food Control. (2021) 125:107962. doi: 10.1016/j.foodcont.2021.107962

[21] Zhou X Sun J Tian Y Yao K Xu M. Detection of heavy metal lead in lettuce leaves based on fluorescence hyperspectral technology combined with deep learning algorithm. Spectrochim Acta A Mol Biomol Spectrosc. (2022) 266:120460. doi: 10.1016/j.saa.2021.120460, PMID: 34637985

[22] Chu H Zhang C Wang M Gouda M Wei X He Y. Hyperspectral imaging with shallow convolutional neural networks (SCNN) predicts the early herbicide stress in wheat cultivars. J Hazard Mater. (2022) 421:126706. doi: 10.1016/j.jhazmat.2021.126706, PMID: 34325290

[23] Yang D Jiang J Jie Y Li Q Shi T. Detection of the moldy status of the stored maize kernels using hyperspectral imaging and deep learning algorithms. Int J Food Prop. (2022) 25:170–86. doi: 10.1080/10942912.2022.2027963

[24] Furbank RT Silva-Perez V Evans JR Condon AG Estavillo GM He W. Wheat physiology predictor: predicting physiological traits in wheat from hyperspectral reflectance measurements using deep learning. Plant Methods. (2021) 17:108. doi: 10.1186/s13007-021-00806-6, PMID: 34666801 PMC8527791

[25] Liang G-C Ouyang Y-C Dai S-M. Detection and classification of Rice infestation with Rice leaf folder (Cnaphalocrocis medinalis) using hyperspectral imaging techniques. Remote Sens. (2021) 13:4587. doi: 10.3390/rs13224587

[26] Weng S Han K Chu Z Zhu G Liu C Zhu Z. Reflectance images of effective wavelengths from hyperspectral imaging for identification of fusarium head blight-infected wheat kernels combined with a residual attention convolution neural network. Comput Electron Agric. (2021) 190:106483. doi: 10.1016/j.compag.2021.106483

[27] He W He H Wang F Wang S Lyu R. Non-destructive detection and recognition of pesticide residues on garlic chive (*Allium tuberosum*) leaves based on short wave infrared hyperspectral imaging and one-dimensional convolutional neural network. Food Measure. (2021) 15:4497–507. doi: 10.1007/s11694-021-01012-7

[28] Wang C Liu B Liu L Zhu Y Hou J Liu P. A review of deep learning used in the hyperspectral image analysis for agriculture. Artif Intell Rev. (2021) 54:5205–53. doi: 10.1007/s10462-021-10018-y

[29] Lammertyn J Peirs A de J Nicolaï B. Light penetration properties of NIR radiation in fruit with respect to non-destructive quality assessment. Postharvest Biol Technol. (2000) 18:121–32. doi: 10.1016/S0925-5214(99)00071-X

[30] Arink M Khan HA Polder G. Light penetration properties of visible and NIR radiation in tomatoes applied to non-destructive quality assessment. Eng Proc. (2021) 9:18. doi: 10.3390/engproc2021009018

[31] Manley M. Near-infrared spectroscopy and hyperspectral imaging: non-destructive analysis of biological materials. Chem Soc Rev. (2014) 43:8200–14. doi: 10.1039/C4CS00062E, PMID: 25156745

[32] Burger J Geladi P. Hyperspectral NIR image regression part I: calibration and correction. J Chemom. (2005) 19:355–63. doi: 10.1002/cem.938

[33] Reddy H Dinakaran S Srisudharson PN Ghosh S Banji D. Near infra red spectroscopy- an overview. Int J ChemTech Res. (2011) 3:825–36.

[34] Barnes RJ Dhanoa MS Lister SJ. Standard normal variate transformation and de-trending of near-infrared diffuse reflectance spectra. Appl Spectrosc. (1989) 43:772–7. doi: 10.1366/0003702894202201

[35] Fearn T Riccioli C Garrido-Varo A Guerrero-Ginel JE. On the geometry of SNV and MSC. Chemom Intell Lab Syst. (2009) 96:22–6. doi: 10.1016/j.chemolab.2008.11.006

[36] Mahynski N. PyChemAuth. (2023). Available at: https://zenodo.org/badge/latestdoi/331207062

[37] Ali M. PyCaret. (2024) Available at: https://www.pycaret.org (Accessed November 29, 2024).

[38] Pedregosa F Varoquaux G Gramfort A Michel V Thirion B Grisel O. Scikit-learn: machine learning in Python. J Mach Learn Res. (2011) 12:2825–30. doi: 10.48550/arXiv.1201.0490

[39] Kingma DP Ba J. Adam: a method for stochastic optimization. 3rd International Conference for Learning Representations, San Diego. (2015).

[40] Ramachandran P Zoph B Le QV. Searching for activation functions (2017). doi: 10.48550/arXiv.1710.05941

[41] Sokolova M Lapalme G. A systematic analysis of performance measures for classification tasks. Inf Process Manag. (2009) 45:427–37. doi: 10.1016/j.ipm.2009.03.002

[42] ElMasry G Sun D-W Allen P. Near-infrared hyperspectral imaging for predicting colour, pH and tenderness of fresh beef. J Food Eng. (2012) 110:127–40. doi: 10.1016/j.jfoodeng.2011.11.028

[43] Barbin D Elmasry G Sun D-W Allen P. Near-infrared hyperspectral imaging for grading and classification of pork. Meat Sci. (2012) 90:259–68. doi: 10.1016/j.meatsci.2011.07.011, PMID: 21821367

[44] Weesepoel Y Alewijn M Daniels F Baart A Müller-Maatsch J Simsek-Senel G. Towards the universal assessment of dietary intake using spectral imaging solutions KIT Publishers. 5th International Conference on Optical Characterization of Materials, Karlsruhe, Germany: Conference Proceedings. (2021).

[45] Esfahani NS Enkatesan E Regentova EE Taghva K Trabia M. Food recognition improvement by using hyper-spectral imagery. Int J Adv Comput Res. (2021) 11:23–50. doi: 10.19101/IJACR.2021.1152006

[46] ElMasry M Nakauchi S. Image analysis operations applied to hyperspectral images for non-invasive sensing of food quality – a comprehensive review. Biosyst Eng. (2016) 142:53–82. doi: 10.1016/j.biosystemseng.2015.11.009

[47] Benes E Gere A Fodor M. Predicting macronutrients and energy content of snack products using FT-NIR analysis and chemometric techniques. J Food Eng. (2020) 280:109954. doi: 10.1016/j.jfoodeng.2020.109954

[48] Popescu M-C Balas VE Perescu-Popescu L Mastorakis N. Multilayer perceptron and neural networks. WSEAS Trans Cir Sys. (2009) 8:579–88. doi: 10.5555/1639537.1639542

[49] Curteanu S Cartwright H. Neural networks applied in chemistry. I. Determination of the optimal topology of multilayer perceptron neural networks. J Chemom. (2011) 25:527–49. doi: 10.1002/cem.1401

[50] Ariana DP Lu R. Hyperspectral waveband selection for internal defect detection of pickling cucumbers and whole pickles. Comput Electron Agric. (2010) 74:137–44. doi: 10.1016/j.compag.2010.07.008

[51] Netherlands Nutrition Center NEVO Nederlandse Voedingmiddelen Tabel 2010 (in English: Dutch food composition table). (2019). Available at: http://nevo-online.rivm.nl (Accessed October 8, 2022).

