# Supplementary material for: The potential of short-wave infrared hyperspectral imaging and deep learning for dietary assessment: a prototype on predicting closed sandwiches fillings
Source: Front Nutr. 2025 Jan 15;11:1520674. doi: 10.3389/fnut.2024.1520674 (PMC11784147; doi:10.3389/fnut.2024.1520674)
Supplement: Supplementary file 1 [file Table_1.docx]

**Table S1:** Purchasing details of the products used for assembly of the sandwiches.

| **Product** | **Image** | **URL** |
| --- | --- | --- |
| White bread (Albert Heijn brand) | 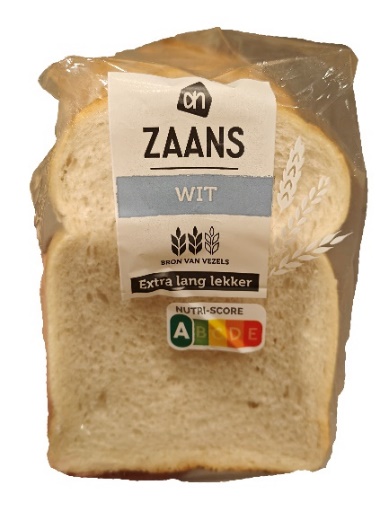 | https://www.ah.nl/producten/product/wi582993/ah-extra-lang-lekker-zaans-wit-half |
| Whole wheat bread (Albert Heijn brand) | 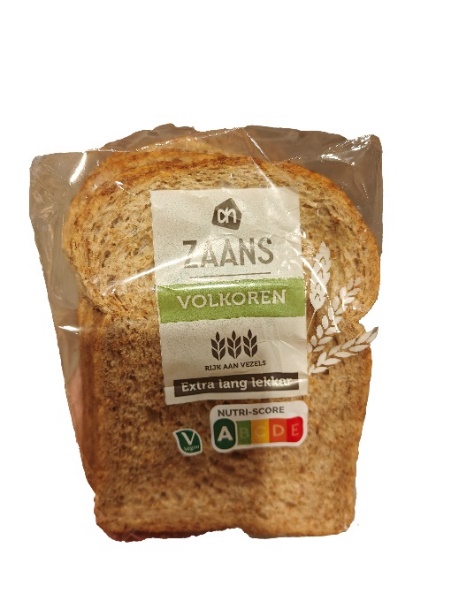 | https://www.ah.nl/producten/product/wi582991/ah-extra-lang-lekker-zaans-volkoren-half |
| Butter (Albert Heijn brand, 50% full fat butter, with plant-based oils to increase spreadability) | 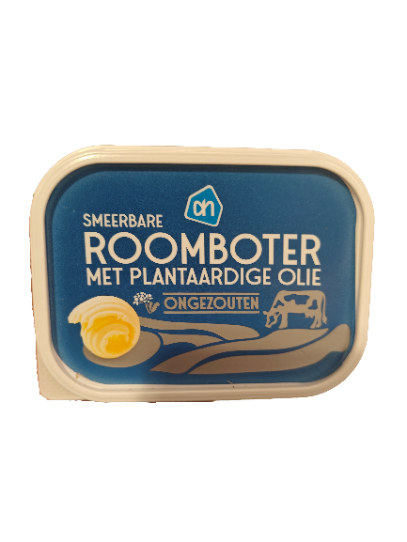 | https://www.ah.nl/producten/product/wi429611/ah-roomboter-met-plantaardige-olie-ongezout |
| Jelly (Hero, strawberry flavor) | 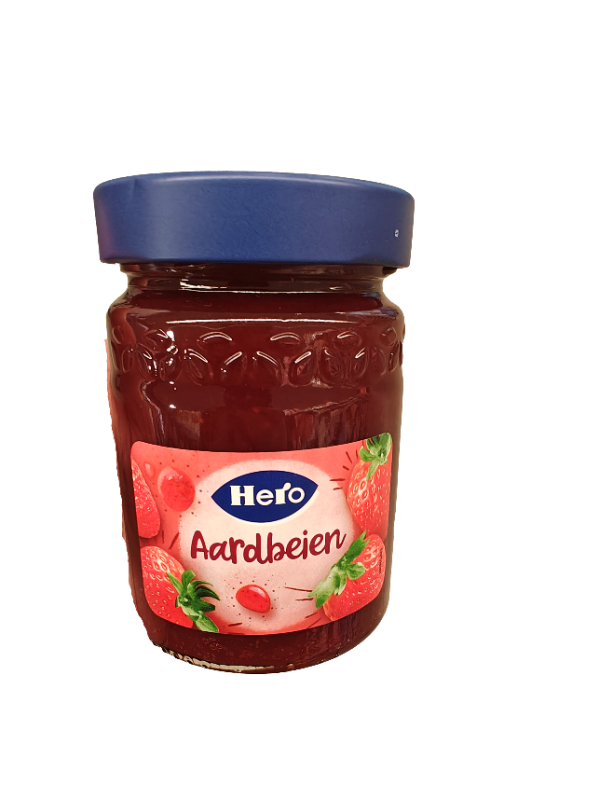 | https://www.ah.nl/producten/product/wi532212/hero-fruitspread-aardbeien |
| Low sugar jelly (Hero, strawberry flavor, reduced sugar) | 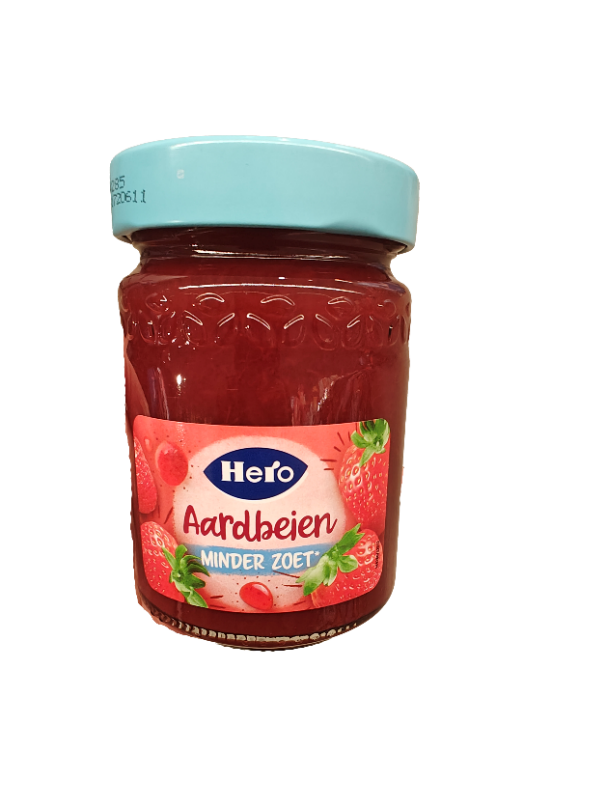 | https://www.ah.nl/producten/product/wi532216/hero-jam-minder-zoet-aardbeien |
| Mature cheese (Albert Heijn, Goudse 48+) | 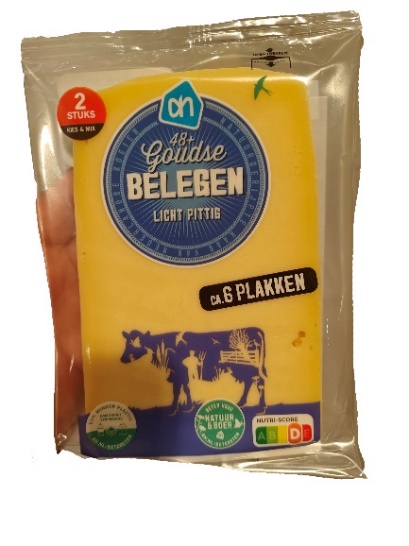 | https://www.ah.nl/producten/product/wi2594/ah-goudse-belegen-48-plakken |
| Low fat mature cheese (Albert Heijn, Goudse 30+), | 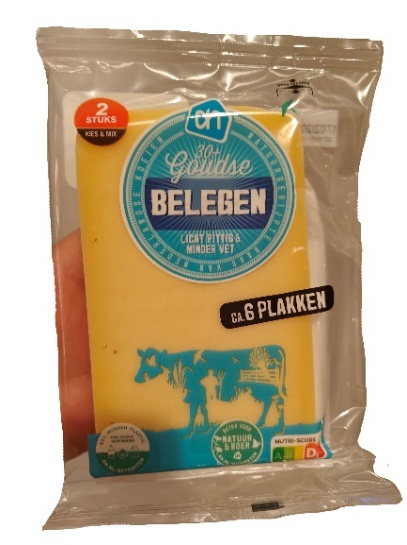 | https://www.ah.nl/producten/product/wi136086/ah-goudse-belegen-30-plakken |
| Peanut butter (Albert Heijn Bio | 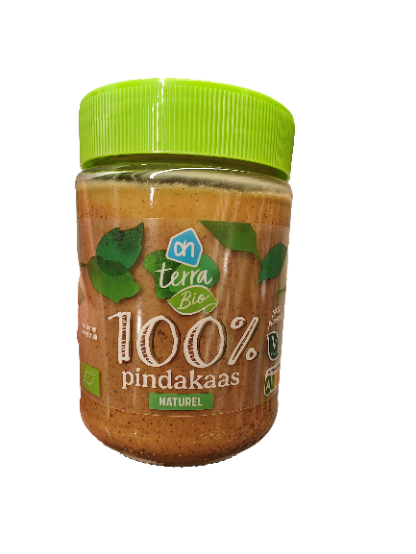 | https://www.ah.nl/producten/product/wi564839/ah-terra-plantaardig-biologisch-100-pindakaas |
| Chocolate sprinkles (De Ruijter, Milk Chocolate). | 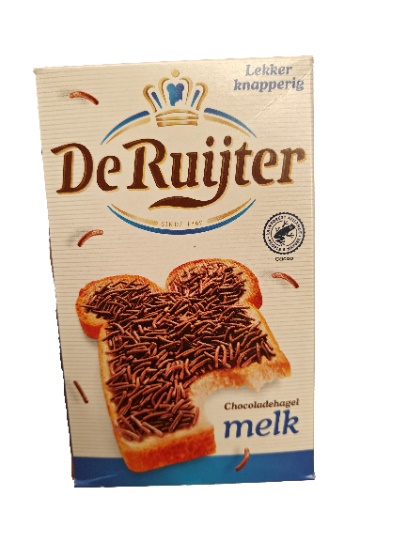 | https://www.ah.nl/producten/product/wi493644/de-ruijter-chocoladehagel-melk |
