# Supplementary material for: The potential of short-wave infrared hyperspectral imaging and deep learning for dietary assessment: a prototype on predicting closed sandwiches fillings
Source: Front Nutr. 2025 Jan 15;11:1520674. doi: 10.3389/fnut.2024.1520674 (PMC11784147; doi:10.3389/fnut.2024.1520674)
Supplement: Supplementary file 3 [file Table_3.docx]

**Table S3:** Confusion matrix results for the best performing pycaret models. This was a Linear Regression model for bread, a linear regression model for butter, and a ridge classifier for fillings.

|  | | | Predicted | | | | | | | | | |
| --- | --- | --- | --- | --- | --- | --- | --- | --- | --- | --- | --- | --- |
|  |  |  | Bread | | Butter | | Filling | | | | | |
|  |  |  | White | Whole Wheat | No | Yes | Mature cheese | Low fat mature cheese | Jelly | Low sugar jelly | Peanut butter | Chocolate sprinkles |
| Ground truth | Bread | White | **0.76** | 0.24 |  |  |  |  |  |  |  |  |
|  |  | Whole wheat | 0.30 | **0.70** |  |  |  |  |  |  |  |  |
|  | Butter | No |  |  | **0.56** | 0.44 |  |  |  |  |  |  |
|  |  | Yes |  |  | 0.42 | **0.58** |  |  |  |  |  |  |
|  | Filling | Mature cheese |  |  |  |  | **0.28** | 0.13 | 0.17 | 0.07 | 0.15 | 0.21 |
|  |  | Low fat mature cheese |  |  |  |  | 0.27 | **0.15** | 0.09 | 0.06 | 0.21 | 0.23 |
|  |  | Jelly |  |  |  |  | 0.18 | 0.10 | **0.24** | 0.09 | 0.11 | 0.28 |
|  |  | Low sugar jelly |  |  |  |  | 0.19 | 0.14 | 0.17 | **0.11** | 0.13 | 0.26 |
|  |  | Peanut butter |  |  |  |  | 0.15 | 0.12 | 0.09 | 0.07 | **0.33** | 0.24 |
|  |  | Chocolate sprinkles |  |  |  |  | 0.11 | 0.08 | 0.12 | 0.04 | 0.10 | **0.56** |
