# Supplementary material for: The potential of short-wave infrared hyperspectral imaging and deep learning for dietary assessment: a prototype on predicting closed sandwiches fillings
Source: Front Nutr. 2025 Jan 15;11:1520674. doi: 10.3389/fnut.2024.1520674 (PMC11784147; doi:10.3389/fnut.2024.1520674)
Supplement: Supplementary file 4 [file Table_4.docx]

**Table S4:** Confusion matrix results for the MLP models.

|  | | | Predicted | | | | | | | | | |
| --- | --- | --- | --- | --- | --- | --- | --- | --- | --- | --- | --- | --- |
|  |  |  | Bread | | Butter | | Filling | | | | | |
|  |  |  | White | Whole Wheat | No | Yes | Mature cheese | Low fat mature cheese | Jelly | Low sugar jelly | Peanut butter | Chocolate sprinkles |
| Ground truth | Bread | White | **0.81** | 0.19 |  |  |  |  |  |  |  |  |
|  |  | Whole wheat | 0.21 | **0.79** |  |  |  |  |  |  |  |  |
|  | Butter | No |  |  | **0.60** | 0.40 |  |  |  |  |  |  |
|  |  | Yes |  |  | 0.41 | **0.59** |  |  |  |  |  |  |
|  | Filling | Mature cheese |  |  |  |  | **0.26** | 0.11 | 0.18 | 0.15 | 0.17 | 0.13 |
|  |  | Low fat mature cheese |  |  |  |  | 0.18 | **0.14** | 0.19 | 0.19 | 0.15 | 0.15 |
|  |  | Jelly |  |  |  |  | 0.18 | 0.07 | **0.28** | 0.21 | 0.14 | 0.13 |
|  |  | Low sugar jelly |  |  |  |  | 0.23 | 0.12 | 0.19 | **0.17** | 0.16 | 0.14 |
|  |  | Peanut butter |  |  |  |  | 0.11 | 0.18 | 0.16 | 0.17 | **0.23** | 0.14 |
|  |  | Chocolate sprinkles |  |  |  |  | 0.08 | 0.07 | 0.22 | 0.17 | 0.10 | **0.37** |
